# Effectiveness and safety of concomitant use of direct oral anticoagulants and antiarrhythmic drugs: a systematic review of observational studies

**DOI:** 10.1007/s00228-025-03883-x

**Published:** 2025-07-16

**Authors:** Fabian Maximilian Meinert, Jenny Dimakos, Thomas Günther Riemer, Antonios Douros

**Affiliations:** 1 Institute of Clinical Pharmacology and Toxicology, Charité - Universitätsmedizin Berlin, Charité Campus Mitte, Luisenstrasse 7, 10117 Berlin, Germany; 2 Department of Medicine, McGill University, Montreal, QC Canada; 3 Department of Epidemiology, Biostatistics and Occupational Health, McGill University, Montreal, QC Canada; 4 School of Epidemiology and Public Health, University of Ottawa, Ottawa, ON Canada

**Keywords:** Direct oral anticoagulants, Antiarrhythmic drugs, Pharmacoepidemiology, Ischemic stroke, Bleeding, Systematic review

## Abstract

**Introduction:**

Concomitant use of antiarrhythmic drugs (AAs) may affect the effectiveness and safety of direct oral anticoagulants (DOACs) through pharmacokinetic interactions and other factors. Our systematic review aimed to provide an in-depth methodological assessment and synthesis of the available real-world evidence in the area.

**Methods:**

We systematically searched MEDLINE/PubMed and EMBASE from January 2011 to October 2024 for observational studies assessing the effectiveness (risk of stroke) and safety (risk of major bleeding) associated with concomitant use of DOACs and AAs. We assessed the risk of bias using the Risk Of Bias In Non-randomized Studies of Interventions (ROBINS-I) tool.

**Results:**

We identified 17 relevant studies including overall 2,613,693 patients. For stroke, all six studies showed no increased risk associated with concomitant use of DOACs and AAs. For major bleeding, seven studies showed an increased risk associated with concomitant use of DOACs and AAs (up to 187%), four studies showed heterogeneous results depending on the specific AA, and six studies showed no increased risk. When considering only higher-quality studies (*n* = 6), there was no association with the risk of stroke (*n* = 3). There were associations with an increased risk of major bleeding for concomitant use of DOACs and diltiazem (*n* = 2) or verapamil (*n* = 1), while findings for concomitant use of DOACs and amiodarone were inconsistent (*n* = 3).

**Conclusions:**

Based on the synthesis of higher-quality real-world evidence, concomitant use of AAs does not seem to impact the effectiveness of DOACs. Findings on safety possibly depend on the specific AA, with diltiazem showing the highest risk.

**Supplementary information:**

The online version contains supplementary material available at 10.1007/s00228-025-03883-x.

## Introduction

Direct oral anticoagulants (DOACs) have shown similar efficacy in preventing ischemic stroke and treating venous thromboembolism (VTE) and improved safety regarding the risk of bleeding compared to vitamin K antagonists (VKAs) [1, 2]. However, their effectiveness and safety is less clear in clinically important subgroups including patients with severe kidney disease [3], chronic liver disease [4], or those who are concomitantly treated with antiarrhythmic drugs.

The effectiveness and safety of DOACs may differ in patients concomitantly treated with antiarrhythmic drugs for various reasons. First, antiarrhythmic drugs are commonly used for rhythm and rate control in non-valvular atrial fibrillation (NVAF) [5, 6], especially among patients with more permanent patterns of arrhythmia. Since such patterns may be indicative of more severe disease and constitute a strong risk factor of stroke [7], patients treated with antiarrhythmic drugs could have a higher baseline risk of adverse clinical outcomes than the overall NVAF population. Second, some antiarrhythmic drugs can pharmacokinetically interact with DOACs via inhibiting the cytochrome P450 3A4 (CYP3A4) enzyme and the permeability glycoprotein (p-pg) transporter [8–10]. These interactions could affect the effectiveness and safety of DOACs.

Post hoc analyses of randomized controlled trials (RCTs) explored the clinical effects of concomitant use of DOACs and antiarrhythmic drugs but were limited due to small sample sizes, confounding bias, and exposure misclassification [11, 12]. Several observational studies attempted to fill this knowledge gap [13–17]. However, their findings were inconsistent and some were possibly affected by important methodological limitations such as information bias and selection bias. Given that the few existing systematic reviews used outdated tools for quality assessment and did not include the more recently published studies [18, 19], there is a need to critically assess the quality of the available observational evidence to determine its relevance for clinical practice. To this end, we systematically reviewed observational studies on the effectiveness and safety of concomitant use of DOACs and antiarrhythmic drugs, thereby thoroughly assessing the risk of bias of the included studies.

## Methods

### Search strategy

This systematic review was conducted and reported using the Preferred Reporting Items for Systematic Reviews and Meta-Analysis (PRISMA). The protocol was pre-registered in PROSPERO (ID: CRD42024521835). We systematically searched MEDLINE/PubMed and EMBASE from January 1, 2011 to October 3, 2024. The start date was chosen because it was the date when dabigatran was approved as the first DOAC for the prevention of ischemic stroke in patients with NVAF, which is the most common indication for DOAC use. We developed a detailed search algorithm with keywords reflecting concomitant use of any DOAC and any antiarrhythmic drug (eTable 1). Additionally, we scanned the bibliography of included studies and previously published systematic reviews of observational studies for further references. Duplicate publications from the same study were removed. We only included publications written in English.

### Types of included studies

We included observational studies (cohort, case–control, nested case–control, and case-only studies). We excluded (1) RCTs, (2) cross-sectional studies because of their temporal ambiguity since they do not establish a temporal sequence between exposure and outcome, (3) case reports and case series due to the lack of a comparator group, (4) reviews, commentaries, editorials and letters to the editor, as they do not provide empirical data, (5) pharmacokinetic studies because they do not assess clinical outcomes, and (6) abstracts and conference proceedings as they do not contain sufficient information to fully assess the quality of the study.

### Populations and exposures

We did not impose any restrictions with respect to the underlying population, that is the indication for DOAC use (e.g., NVAF, VTE). Patients in the included studies had to be concomitant users of a DOAC and an antiarrhythmic drug to be in the ‘exposed’ group. Given the different approaches used in observational studies that assess the effects of concomitant use of multiple drugs with respect to the reference group [20], potential reference groups included (1) use of a DOAC only, (2) concomitant use of a DOAC and a non-antiarrhythmic drug, (3) concomitant use of a VKA and an antiarrhythmic drug, or (4) concomitant use of DOAC and a different antiarrhythmic drug than the one included in the ‘exposed’ group.

### Outcomes

The primary effectiveness outcome was stroke or systemic embolism (SE). The primary safety outcome was major bleeding. The secondary outcome was all-cause mortality. We included a study if at least one of the following effect estimates were provided: relative risk, odds ratio (OR), hazard ratio (HR), incidence rate ratio, or when sufficient data were provided to calculate any one of these measures.

### Data extraction

Data were independently extracted by two reviewers (FMM/JD) using Excel. First, we extracted study characteristics including data source, study design, study period, indication for use of DOACs, size of the study cohort, statistical model, main comparisons, and main outcomes, along with the relevant effect estimates. Second, we extracted clinical data including age, sex, baseline bleeding risk, comorbidity burden, prior stroke, prior major bleeding, and chronic kidney disease. Disagreements were resolved by either consensus or a third reviewer (AD).

### Assessment of risk of bias

Two reviewers (FMM/JD) independently assessed the risk of bias of included studies using the Risk Of Bias In Non-randomized Studies of Interventions (ROBINS-I) tool for observational studies [21]. The ROBINS-I includes the following domains regarding the risk of bias: (1) bias due to confounding, (2) bias in selection of participants into study, (3) bias in classification of interventions, (4) bias due to departures from intended interventions, (5) bias due to missing data, (6) bias in measurement of outcomes, and (7) bias in selection of the reported results. Given the different potential forms of bias in selection of participants into study (from now on simply selection bias for brevity), especially in studies on drug-drug interactions, we followed a ‘customized’ approach. We initially created a list of forms of selection bias (exclusion of persons or person-time after cohort entry, depletion of susceptibles due to inclusion of prevalent users, depletion of susceptibles due to design aspects, immortal time bias, informative censoring). Then, we based our assessment for this domain of ROBINS-I using the overall number of different forms of selection bias affecting each study (≥ 3 forms of selection bias: critical risk; 2 forms of selection bias: serious risk; 1 form of selection bias: moderate risk; 0 forms of selection bias: low risk). Each study was assigned to an overall low, moderate, serious, or critical risk of bias, as determined by the domain with the highest risk of bias. Moreover, the presence of biases that are specific to pharmacoepidemiology and especially to the pharmacoepidemiology of drug-drug interactions and are not fully covered by ROBINS-I (e.g., immortal time bias, depletion of susceptibles bias, time-window bias) was also assessed [20]. Disagreements were resolved by consensus or, if necessary, with a third author (AD).

### Meta-analyses of higher-quality studies

After the completion of the assessment of the risk of bias, we decided to conduct post-hoc meta-analyses for associations with at least three studies at an overall moderate risk of bias. Meta-analyses were conducted using random-effects models.

## Results

**Fig. 1:**
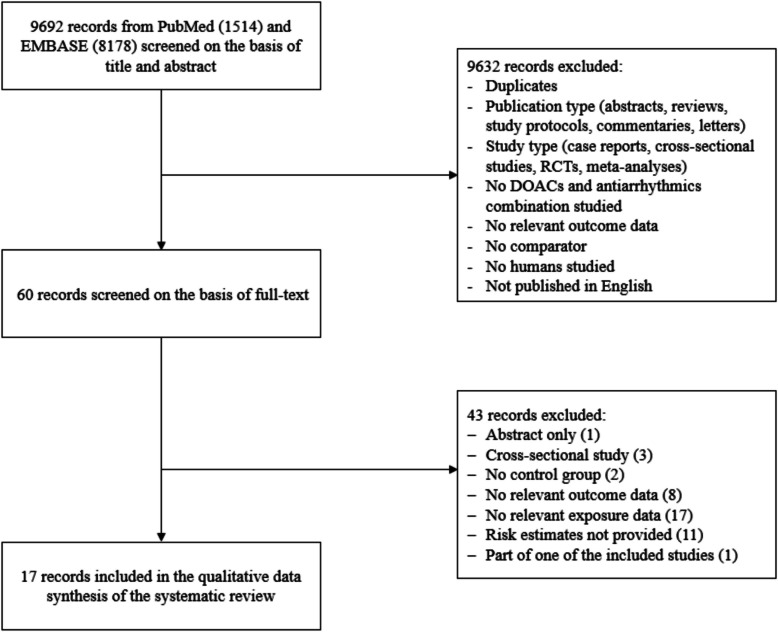
Flowchart illustrating the selection of included studies. Abbreviations: RCTs, randomized controlled trials; DOACs, direct oral anticoagulants

The systematic literature search revealed 9692 studies overall. We excluded 9632 studies during screening on the basis of title and abstract; the remaining 60 studies underwent full-text review, and 43 of them were subsequently excluded for reasons outlined in Fig. 1, leaving 17 studies to be considered in the systematic review. The studies were published between 2017 and 2024 and included overall 2,613,693 patients (eTable 2). Nine studies were conducted in North America [13, 17, 22–28], four studies in Asia [14, 29–31], and four studies in Europe [32–35].

Twelve studies used healthcare claims data [13, 14, 17, 22, 24–29, 32, 35], two studies used nationwide registries [33, 34], and three studies used electronic health records (eTable 2) [23, 30, 31]. Three studies applied nested case–control designs [14, 24, 25] and 14 studies applied cohort designs [13, 17, 22, 23, 26–35]. AF was explicitly mentioned as an inclusion criterion in 13 studies [13, 14, 17, 22–26, 28–30, 33, 34]; the other four studies did not specify the indication for DOAC use [27, 31, 32, 35]. Five studies included patients adding on an antiarrhythmic drug while on a DOAC [14, 17, 26, 28, 31], three studies included patients adding on a DOAC while on an antiarrhythmic drug[13, 22, 32], while the temporal order of the two drugs was not specified in nine studies [23–25, 27, 29, 30, 33–35]. Eight studies compared concomitant use of DOACs and antiarrhythmic drugs versus use of DOACs alone [14, 22–25, 29, 30, 32], six studies compared concomitant use of DOACs and antiarrhythmic drugs versus concomitant use of DOACs and non-antiarrhythmic drugs [13, 26–28, 31, 35], two studies compared concomitant use of DOACs and antiarrhythmic drugs versus concomitant use of VKAs and antiarrhythmic drugs [33, 34], and one study compared concomitant use of DOACs and antiarrhythmic drugs versus concomitant use of DOACs and other antiarrhythmic drugs [17]. Selected patient characteristics in the included studies can be found in eTable 3.

### Concomitant use of DOACs and antiarrhythmic drugs versus use of DOACs alone

Eight studies compared concomitant use of any or specific DOACs and antiarrhythmic drugs versus use of any or specific DOACs alone regarding the risk of major bleeding (Table 1) [14, 22–25, 29, 30, 32]. Most comparisons yielded increased risks, with the strongest increases being observed for DOACs plus verapamil versus DOACs (OR, 1.56; 95% CI, 1.02–2.39) [14] and for DOACs plus diltiazem versus DOACs (HR, 1.56; 95% CI, 1.15–2.12) [23]. Two studies assessed the risk of stroke/SE and all-cause mortality showing no differences [30, 32].

**Table 1:** Main findings of the included studies

Study	Comparisons	Effect estimates (95% CI)
	**DOACs + PK-AA vs DOACs**	
Chang [29]	DOACs + amiodarone vs DOACs DOACs + diltiazem vs DOACs DOACs + dronedarone vs DOACs DOACs + verapamil vs DOACs	**Major bleeding:** RR, 1.37 (1.27–1.46) **Major bleeding:** RR, 0.94 (0.87–1.01) **Major bleeding:** RR, 0.89 (0.75–1.07) **Major bleeding:** RR, 1.12 (0.98–1.29)
Chiou [30]	RIVA + amiodarone, propafenone, or dronedarone vs RIVA	**Major bleeding:** HR, 1.11 (0.82–1.49) **Stroke/SE:** HR, 0.90 (0.52–1.56) **All-cause mortality:** HR, 0.73 (0.50–1.07)
Gandhi [22]	APIXA + dronedarone vs APIXA DABI + dronedarone vs DABI RIVA + dronedarone vs RIVA	**Major bleeding:** IRR, 0.74 (0.44–1.25) **Major bleeding:** IRR, 1.33 (1.01–1.75) **Major bleeding:** IRR, 1.48 (1.16–1.90)
Gronich [14]	DOACs + amiodarone vs DOACs DOACs + diltiazem vs DOACs DOACs + dronedarone vs DOACs DOACs + verapamil vs DOACs	**Major bleeding:** OR, 1.21 (0.96–1.52) **Major bleeding:** OR, 1.02 (0.37–2.82) **Major bleeding:** OR, 1.48 (0.64–3.43) **Major bleeding:** OR, 1.56 (1.02–2.39)
Grymonprez [32]	DOACs + amiodarone vs DOACs DOACs + diltiazem vs DOACs DOACs + verapamil vs DOACs	**Major bleeding:** HR, 1.27 (1.21–1.34) **Stroke/SE:** HR, 1.00 (0.92–1.09) **All-cause mortality:** HR, 1.10 (1.05–1.15) **Major bleeding:** HR, 1.28 (1.13–1.46) **Stroke/SE:** HR, 1.00 (0.81–1.24) **All-cause mortality:** HR, 0.94 (0.84–1.05) **Major bleeding:** HR, 1.36 (1.03–1.80) **Stroke/SE:** HR, 0.80 (0.51–1.24) **All-cause mortality:** HR, 0.95 (0.73–1.23)
Shurrab [24]	DOAC + diltiazem vs DOACs	**Major bleeding:** OR, 1.37 (1.08–1.73)
Shurrab [25]	DOACs + amiodarone vs DOACs	**Major bleeding:** OR, 1.53 (1.24–1.89)
Xu [23]	DOACs + diltiazem vs DOACs	**Major bleeding:** HR, 1.56 (1.15–2.12)
	**DOACs + PK-AA vs DOACs + non-AA**	
Hill [26]	DOACs + amiodarone vs DOACs + metoprolol DOACs + diltiazem vs DOACs + amlodipine DOACs + verapamil vs DOACs + amlodipine	**Major bleeding:** HR, 0.77 (0.61–0.97) **Major bleeding:** HR, 0.99 (0.85–1.15) **Major bleeding:** HR, 1.32 (0.88–1.98)
Komatsu [31]	DOACs + verapamil vs DOACs + bepridil	**Hemorrhage:** HR, 2.87 (1.17–7.07)
Pham [13]	APIXA + verapamil or diltiazem vs APIXA + amlodipine DABI + verapamil or diltiazem vs DABI + amlodipine RIVA + verapamil or diltiazem vs RIVA + amlodipine APIXA + verapamil or diltiazem vs APIXA + metoprolol DABI + verapamil or diltiazem vs DABI + metoprolol RIVA + verapamil or diltiazem vs RIVA + metoprolol	**Major bleeding:** HR, 1.57 (0.35–7.16) **Major bleeding:** HR, 2.27 (0.97–5.29) **Major bleeding:** HR, 1.23 (0.65–2.35) **Major bleeding:** HR, 1.46 (0.33–6.41) **Major bleeding:** HR, 3.32 (1.54–7.16) **Major bleeding:** HR, 0.99 (0.50–1.98)
Ray [28]	DOACs + diltiazem vs DOACs + metoprolol	**Major bleeding:** HR, 1.21 (1.13–1.29) **Stroke/SE:** HR, 0.87 (0.74–1.03)
Teshima [27]	APIXA + diltiazem or verapamil vs APIXA + metoprolol DABI + diltiazem or verapamil vs DABI + metoprolol RIVA + diltiazem or verapamil vs RIVA + metoprolol	**Major bleeding:** HR, 1.18 (0.81–1.72) **All-cause mortality:** HR, 0.79 (0.43–1.46) **Major bleeding:** HR, 1.07 (0.86–1.38) **All-cause mortality:** HR, 1.05 (0.76–1.45) **Major bleeding:** HR, 0.74 (0.35–1.57) **All-cause mortality:** HR, 1.09 (0.47–2.50)
Wong [35]	DOACs + amiodarone vs DOACs + beta-blockers DOACs + diltiazem vs DOACs + beta-blockers DOACs + verapamil vs DOACs + beta-blockers	**Intracranial bleeding:** HR, 1.60 (0.47–5.49) **Gastrointestinal bleeding:** HR, 0.96 (0.55–1.68) **Other bleeding:** HR, 0.61 (0.39–0.96) **Stroke:** HR, 0.69 (0.34–1.42) **All-cause mortality:** HR, 0.96 (0.71–1.31) **Intracranial bleeding:** HR, 0.82 (0.19–3.50) **Gastrointestinal bleeding:** HR, 1.03 (0.67–1.60) **Other bleeding:** HR, 1.02 (0.69–1.50) **Stroke:** HR, 0.74 (0.34–1.58) **All-cause mortality:** HR, 0.78 (0.58–1.05) **Intracranial bleeding:** no exposed events **Gastrointestinal bleeding:** HR, 1.86 (0.88–3.95) **Other bleeding:** HR, 1.06 (0.43–2.62) **Stroke:** HR, 0.97 (0.32–2.93) **All-cause mortality:** HR, 0.68 (0.38–1.22)
	**DOACs + PK-AA vs VKAs + PK-AA**	
Friberg [33]	APIXA + dronedarone vs warfarin + dronedarone	**Major Bleeding:** HR, 0.66 (0.35–1.23) **All-cause mortality:** HR, 1.45 (0.42–5.03)
Fritz Hansson [34]	APIXA + amiodarone vs warfarin + amiodarone	**Major bleeding:** HR, 1.03 (0.76–1.39) **Stroke/SE:** HR, 0.98 (0.58–1.68) **All-cause mortality:** HR, 1.23 (0.96–1.57)
	**DOACs + PK-AA vs DOACs + non-PK-AA**	
Ray [17]	DOACs + amiodarone vs DOACs + flecainide or sotalol	**Major bleeding:** HR, 1.42 (1.27–1.63) **Stroke/SE:** HR, 0.80 (0.62–1.03) **All-cause mortality:** HR, 1.28 (1.15–1.44)

*DOACs*; direct oral anticoagulants, *PK-AA*; pharmacokinetically-interacting antiarrhythmic drugs, *VKAs*; vitamin K antagonists, *APIXA*; apixaban, *DABI*; dabigatran, *RIVA*; rivaroxaban, *SE*; systemic embolism, *CI*; confidence interval, *RR*; risk ratio, *HR*; hazard ratio, *OR*; odds ratio

### Concomitant use of DOACs and antiarrhythmic drugs versus concomitant use of DOACs and non-antiarrhythmic drugs

Six studies compared concomitant use of any or specific DOACs and antiarrhythmic drugs versus concomitant use of any or specific DOACs and non-antiarrhythmic drugs regarding the risk of major or any bleeding (Table 1) [13, 17, 26, 27, 31, 35]. Two studies showed increased risks (HRs, 1.21 & 2.87) [17, 31], one study showed mixed findings depending on the antiarrhythmic drug and the subtype of major bleeding [35], two studies showed no differences in the risk [26, 27], while one study lacked the necessary statistical power [13]. Three studies assessed the risk of stroke/SE and the risk of all-cause mortality, respectively, showing no differences in the risk [17, 27, 35].

### Other comparisons

Two studies compared concomitant use of apixaban and antiarrhythmic drugs (dronedarone or amiodarone) versus concomitant use of VKAs and the same antiarrhythmic drugs regarding the risk of major bleeding showing no differences (Table 1) [33, 34]. Analyses on the risk of stroke/SE and all-cause mortality either lacked the necessary statistical power or showed no differences. One study compared concomitant use of DOACs and amiodarone versus concomitant use of DOACs and flecainide or sotalol, showing a 42% increased risk of major bleeding, no difference in the risk of stroke/SE, and a 28% increased risk of all-cause mortality [17].

### Risk of bias assessment

Based on ROBINS-I, six out of the 17 studies were assigned an overall moderate risk of bias [13, 17, 23, 26, 28, 32], nine studies were assigned an overall serious risk of bias [14, 22, 24, 25, 27, 31, 33–35], and two studies were assigned an overall critical risk of bias (Table 2) [29, 30]. The biases most often responsible for the overall assessment were bias due to confounding and bias in the selection of participants into the study.

**Table 2:** Assessment of risk of bias of the included studies using ROBINS-I

Study	Bias due to confounding	Bias in selection of participants into study	Bias in classification of interventions	Bias due to departures from intended interventions	Bias due to missing data	Bias in measurement of outcomes	Bias in selection of the reported result	Overall
Chang [29]	Moderate	Critical	Critical	NA	NA	Low	Moderate	Critical
Chiou [30]	Moderate	Critical	Moderate	NA	NA	Low	Moderate	Critical
Friberg [33]	Moderate	Serious	Moderate	NA	NA	Low	Moderate	Serious
Fritz Hansson [34]	Moderate	Serious	Low	NA	NA	Low	Moderate	Serious
Gandhi [22]	Moderate	Serious	Low	NA	NA	Low	Moderate	Serious
Gronich [14]	Serious	Moderate	Moderate	NA	NA	Low	Moderate	Serious
Grymonprez [32]	Moderate	Moderate	Moderate	NA	NA	Low	Moderate	Moderate
Hill [26]	Moderate	Moderate	Moderate	NA	NA	Low	Moderate	Moderate
Komatsu [31]	Moderate	Serious	Moderate	NA	NA	Moderate	Moderate	Serious
Pham [13]	Moderate	Moderate	Low	NA	NA	Low	Moderate	Moderate
Ray [17]	Moderate	Low	Low	NA	NA	Low	Moderate	Moderate
Ray [28]	Moderate	Low	Low	NA	NA	Low	Moderate	Moderate
Shurrab [24]	Serious	Serious	Low	NA	NA	Low	Moderate	Serious
Shurrab [25]	Serious	Serious	Low	NA	NA	Low	Moderate	Serious
Teshima [27]	Moderate	Serious	Moderate	NA	NA	Low	Moderate	Serious
Wong [35]	Moderate	Serious	Low	NA	NA	Low	Moderate	Serious
Xu [23]	Moderate	Moderate	Moderate	NA	NA	Low	Moderate	Moderate

*ROBINS-I*; Risk Of Bias In Non-randomized Studies of Interventions, *NA*; not applicable

Regarding bias due to confounding, three studies were assigned a serious risk due their use of a nested case–control design, meaning that confounders were not measured at the time-point of initiation of concomitant use [14, 24, 25]. Moreover, these studies did not use a control precipitant, which can further aggravate confounding in drug-drug interaction studies [20]. The remaining 14 studies were assigned a moderate risk of bias [13, 14, 22–32, 35].

Regarding bias due to the selection of participants into the study, two studies were assigned a critical risk, eight studies were assigned a serious risk [22, 24, 25, 27, 29–31, 33–35], five studies were assigned a moderate risk [13, 14, 23, 26, 32], and two studies were assigned a low risk [17, 28]. The risk of bias in this domain was mostly driven by depletion of susceptibles due to the inclusion of prevalent users and the removal of persons or person-time after cohort entry. A detailed assessment of potential sources of selection bias can be found in eTable 4.

Regarding bias due to the classification of interventions, one study was assigned a critical risk of bias [29]. The study used three-month periods as the analytic time-unit and assumed that a single prescription for the object drug and a single prescription for the precipitant drug during that period guaranteed continuous co-exposure [29]. Eight studies were assigned a moderate risk [14, 23, 26, 27, 30–33] mostly due to the use of overly long grace periods, which can introduce some misclassification of exposure, and eight studies were assigned a low risk (Table 2) [13, 17, 22, 24, 25, 28, 34, 35].

Regarding the other ROBINS-I domains that were applicable for our systematic review, all studies except one were assigned a low risk of bias in the measurement of outcomes [31], while all studies were assigned a moderate risk of bias in the selection of the reported results due to the lack of a publicly available study protocol. Finally, two of the studies using a nested case–control design did not match on duration of follow-up [24, 25], thus possibly introducing time-window bias [36].

### Evidence synthesis of higher-quality studies

**Fig. 2:**
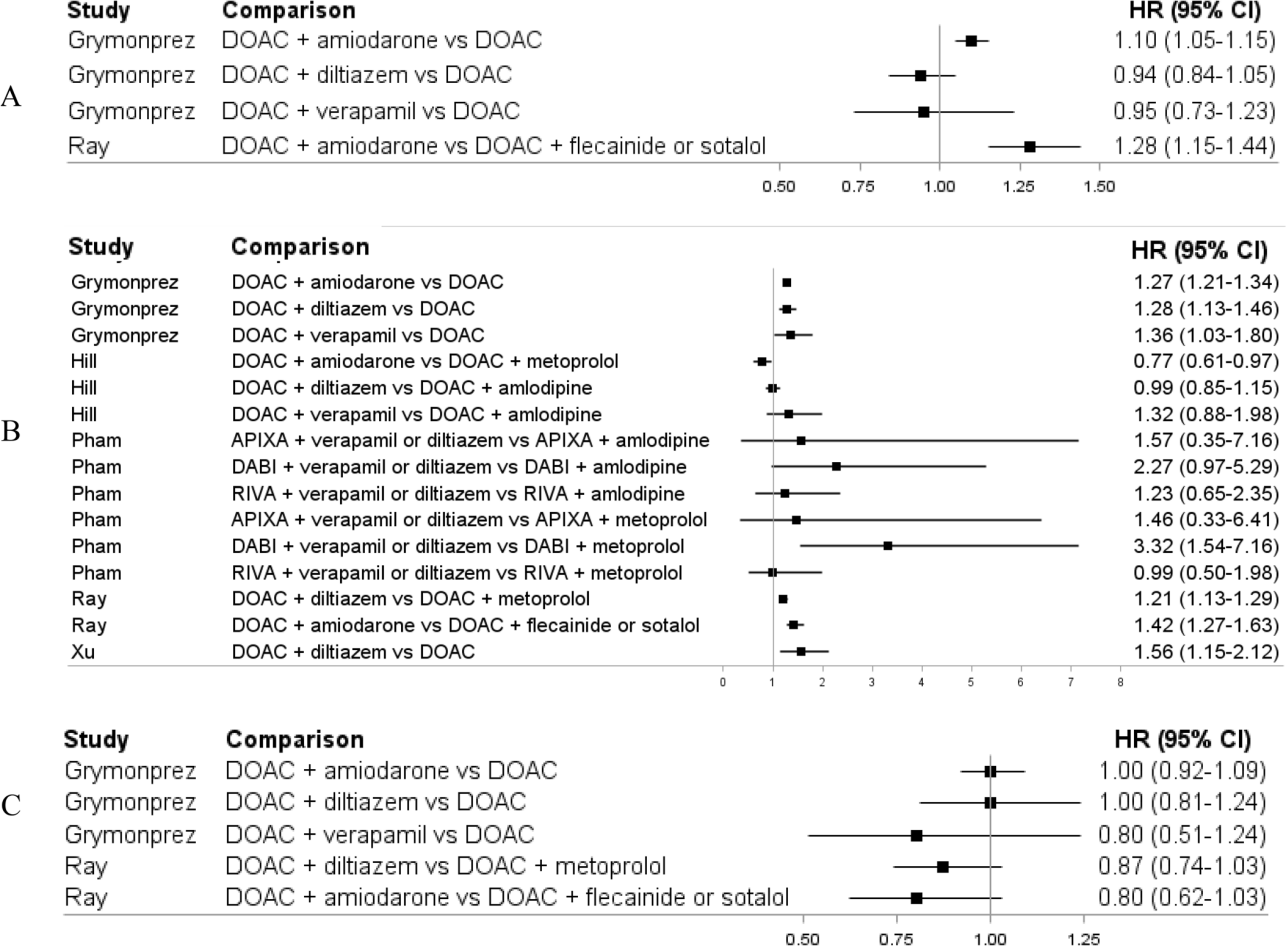
Forest plots summarizing the main findings of higher-quality observational studies assessing the effectiveness and safety of concomitant use of DOACs and antiarrhythmic drugs. Panel (**A**). Ischemic stroke. Panel (**B**). Major bleeding. Panel (**C**). All-cause mortality. Abbreviations: DOAC, direct oral anticoagulant; DABI, dabigatran; RIVA, rivaroxaban; APIXA, apixaban; HR, hazard ratio; CI, confidence interval

There were six higher-quality studies with an overall moderate risk of bias [13, 17, 23, 26, 28, 32]. Two higher-quality studies compared DOACs and antiarrhythmics drugs versus DOACs alone showing increased risks of major bleeding (from 27% with amiodarone to 56% with diltiazem) [23, 32] but no differences in the risk of stroke/SE or all-cause mortality [32]. Three higher-quality studies compared concomitant use of DOACs and antiarrhythmic drugs versus concomitant use of DOACs and non-antiarrhythmic drugs [13, 26, 28]. Regarding the risk of major bleeding, the first study showed heterogenous findings (from 23% decreased risk with amiodarone to 31% numerically increased risk with verapamil) [26], the second study lacked the necessary statistical power [13], and the third study showed a 21% increased risk with diltiazem [28]. There were no associations with the risk of stroke/SE or all-cause mortality [28]. Finally, one higher-quality study comparing concomitant use of DOACs and amiodarone versus concomitant use of DOACs and flecainide or sotalol showed a 42% increased risk of major bleeding, no difference in the risk of stroke/SE, and a 28% increased risk of all-cause mortality [17]. The findings of higher-quality studies are summarized in the forest plots in Fig. 2.

### Meta-analyses of higher-quality studies

**Fig. 3:**
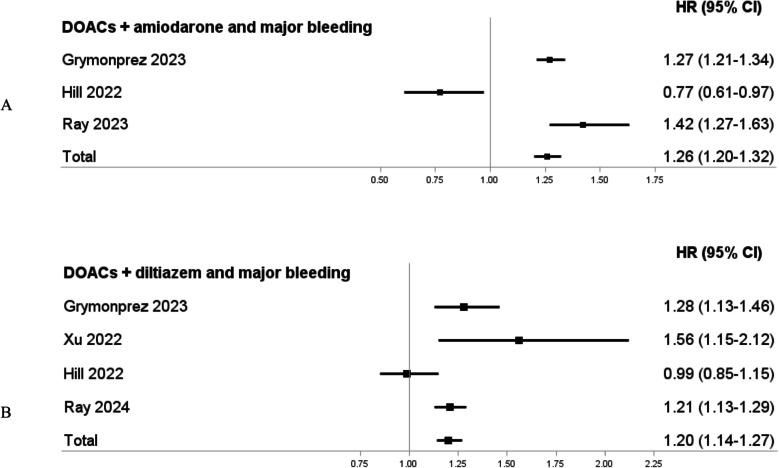
Forest plots showing the findings of meta-analyses for associations assessed by at least three higher-quality observational studies. Panel A. Concomitant use of DOACs and amiodarone and the risk of major bleeding. Panel B. Concomitant use of DOACs and diltiazem and the risk of major bleeding. Abbreviations: DOACs, direct oral anticoagulants; HR, hazard ratio; CI, confidence interval

Concomitant use of DOACs and amiodarone was associated with an increased risk of major bleeding versus different comparators (pooled HR, 1.26; 95% CI, 1.20 to 1.32; *I*^2^ = 90%). Moreover, concomitant use of DOACs and diltiazem was associated with an increased risk of major bleeding versus different comparators (pooled HR, 1.20; 95% CI, 1.14 to 1.27; *I*^2^ = 70%) (Fig. 3).

## Discussion

Our systematic review included 17 observational studies with over two million patients. Concomitant use of DOACs and antiarrhythmics was not associated with a risk of stroke. Regarding major bleeding, there were associations with an up to 56% increased risk when comparing concomitant use of DOACs and antiarrhythmic drugs to use of DOACs alone; these associations were less consistent when comparing to concomitant use of DOACs and non-antiarrhythmic drugs, and they were further attenuated when comparing to concomitant use of VKAs and antiarrhythmic drugs or concomitant use of DOACs and non-interacting antiarrhythmic drugs. Restricting to higher-quality studies corroborated the lack of an association with the risk of stroke. It also suggested that concomitant use of DOACs and antiarrhythmic drugs such as diltiazem and verapamil but not necessarily amiodarone is associated with an increased risk of major bleeding.

Overall, the results of our evidence synthesis are concordant with available pharmacokinetic data. DOACs, with the exception of dabigatran, are CYP3A4 substrates [37]. Several antiarrhythmic drugs such as diltiazem, verapamil, or amiodarone are moderate or weak inhibitors of CYP34A4, and their concomitant use has been shown to lead to increased systemic levels of DOACs (up to 40% with diltiazem, up to 53% with verapamil, and up to 40% with amiodarone) [38]. Therefore, concomitant use of DOACs and certain antiarrhythmic drugs could increase the risk of major bleeding or decrease the risk of stroke.

We observed an increased risk of major bleeding, with diltiazem and verapamil showing more consistent results among higher-quality studies than amiodarone. This seems to reflect the strength of CYP3A4 inhibition of individual antiarrhythmics, since diltiazem and verapamil have been classified as moderate inhibitors, while amiodarone has been classified as weak inhibitor [38]. Of note, there was no association between concomitant use of DOACs and antiarrhythmic drugs and the risk of stroke. One possible explanation is that the effectiveness of DOACs does not depend on drug levels to the same extent as their safety. That being said, the number of studies focusing on the risk of stroke was overall limited; therefore, further studies are needed to corroborate these findings.

Eleven out of the 17 identified observational studies were assessed to be at an overall serious or critical risk of bias. Biases most commonly contributing to this assessment were important residual confounding and different forms of selection bias. Indeed, several studies did not use control precipitants as part of their active comparator, which is a well-established approach to decrease confounding in studies on the clinical effects of drug-drug interactions [20, 39, 40]. In this setting, potential control precipitants may include non-antiarrhythmic cardiovascular drugs or antiarrhythmic drugs not interacting with DOACs. Moreover, selection bias, which was most often introduced in the form of prevalent-user bias, could have been alleviated by considering prior use of the object drug (i.e., DOACs) in the analyses.

Our systematic review has several strengths. First, we provide the first comprehensive synthesis of existing real-world-evidence for an important clinical question. Indeed, prior systematic reviews in the area either focused on post-hoc analyses of RCTs or included a mix of different study types (post hoc analyses of RCTs, observational studies, case reports, or case series). Second, we conducted an in-depth assessment of the methodological quality of the included studies. To this end, we used ROBINS-I, a state-of-the-art tool recommended by the Cochrane group [21]. Third, we classified studies based on their reference group and specifically based on the use or not of a control precipitant. By doing so, we exemplified the importance of control precipitants as part of an active comparator in observational studies on drug-drug-interactions.

Our systematic review also has limitations, all related to the included studies. First, there was considerable methodological heterogeneity between studies, for example with respect to the antiarrhythmic drug of interest, the choice of reference group, the design, the definition of the outcome, or other factors. Therefore, a meta-analysis including all identified studies was not deemed possible or informative. Second, only a small number of higher-quality studies assessed the risk of stroke. Therefore, our conclusions regarding the potential impact of concomitant use of antiarrhythmic drugs on the effectiveness of DOACs needs to be corroborated by future studies. Third, while the number of higher-quality studies assessing the risk of major bleeding was higher, some knowledge gaps remain, especially regarding combinations of specific DOACs and specific antiarrhythmic drugs. Fourth, we did not assess the risk of (recurrent) VTE in our systematic review given the expected low number of studies focusing on anticoagulation for the prevention or treatment of VTE. Indeed, only two of the included studies contained patients with VTE in addition to patients with NVAF. That being said, should studies on the effects of concomitant use of DOACs and antiarrhythmic drugs be conducted among patients with VTE in the future, separate evidence synthesis for this indication will be warranted.

In conclusion, the findings of this systematic review suggest that concomitant use of DOACs and antiarrhythmic drugs does not seem to be associated with the risk of stroke. However, there was an association with an increased risk of major bleeding, especially with diltiazem and verapamil. Future, well-conducted observational studies should corroborate the findings regarding the risk of stroke and fill-in remaining knowledge gaps regarding the risk of major bleeding.

## Supplementary information

Below is the link to the electronic supplementary material.

ESM 1 (DOCX 123 KB)

## Data Availability

No datasets were generated or analysed during the current study.
