# Supplementary material for: Effectiveness and safety of concomitant use of direct oral anticoagulants and antiarrhythmic drugs: a systematic review of observational studies
Source: Eur J Clin Pharmacol. 2025 Jul 16;81(10):1409–19. doi: 10.1007/s00228-025-03883-x (PMC12443910; doi:10.1007/s00228-025-03883-x)
Supplement: Supplementary file 1 — (DOCX 123 KB) [file 228_2025_3883_MOESM1_ESM.docx]

**Content**

[eTable 1. Search strategy 2](#_Toc182999768)

[eTable 2. Selected characteristics of the included studies 3](#_Toc182999769)

[eTable 3. Selected clinical characteristics of patients in the included studies 6](#_Toc182999770)

[eTable 4. Different forms of bias in selection of participants into study 8](#_Toc182999771)

[REFERENCES 9](#_Toc182999772)

# **eTable 1. Search strategy**

| **Database** | **Search algorithm** |
| --- | --- |
| PubMed/MEDLINE | (“anticoagulant” OR “anticoagulants” OR “oral anticoagulant” OR “oral anticoagulants” OR “direct oral anticoagulant” OR “direct oral anticoagulants” OR “doac” OR “doacs” OR “non-vitamin k oral anticoagulant” OR “non-vitamin k oral anticoagulants” OR “noac” OR “noacs” OR “dabigatran” OR “betrixaban” OR “apixaban” OR “rivaroxaban” OR “edoxaban” OR “Xa inhibitor” OR “Xa inhibitors” OR “IIa inhibitor” OR “IIa inhibitors” OR “direct thrombin inhibitor” OR “direct thrombin inhibitors”)  AND  (“antiarrhythmic” OR “antiarrhythmics” OR “propafenone” OR “flecainide” OR “Sotalol” OR “Verapamil” OR “diltiazem” OR “dronedarone” OR “amiodarone” OR “natrium channel blockers” OR “na channel blocker” OR “sodium channel blocker” OR “sodium channel blockers” OR “potassium channel blocker” OR “potassium channel blockers” OR “ k channel blocker” OR “k channel blockers” OR “calcium channel blocker” OR “calcium channel blockers” OR “ca channel blocker” OR “ca channel blockers” OR “calciumantagonist” OR “calciumantagonists” OR “beta blocker” OR “beta blockers” OR “beta receptor blocker” OR “beta receptor blockers” OR “non-dihydropyridine” OR “non-dihydropyridines” OR “phenylethylamine” OR “phenylethylamines”) |
| EMBASE | (“anticoagulant” OR “anticoagulants” OR “oral anticoagulant” OR “oral anticoagulants” OR “direct oral anticoagulant” OR “direct oral anticoagulants” OR “doac” OR “doacs” OR “non-vitamin k oral anticoagulant” OR “non-vitamin k oral anticoagulants” OR “noac” OR “noacs” OR “dabigatran” OR “betrixaban” OR “apixaban” OR “rivaroxaban” OR “edoxaban” OR “Xa inhibitor” OR “Xa inhibitors” OR “IIa inhibitor” OR “IIa inhibitors” OR “direct thrombin inhibitor” OR “direct thrombin inhibitors”)  AND  (“antiarrhythmic” OR “antiarrhythmics” OR “propafenone” OR “flecainide” OR “Sotalol” OR “Verapamil” OR “diltiazem” OR “dronedarone” OR “amiodarone” OR “natrium channel blockers” OR “na channel blocker” OR “sodium channel blocker” OR “sodium channel blockers” OR “potassium channel blocker” OR “potassium channel blockers” OR “ k channel blocker” OR “k channel blockers” OR “calcium channel blocker” OR “calcium channel blockers” OR “ca channel blocker” OR “ca channel blockers” OR “calciumantagonist” OR “calciumantagonists” OR “beta blocker” OR “beta blockers” OR “beta receptor blocker” OR “beta receptor blockers” OR “non-dihydropyridine” OR “non-dihydropyridines” OR “phenylethylamine” OR “phenylethylamines”) |

Abbreviations: DOAC, direct oral anticoagulant; NOAC, non-vitamin K antagonist oral anticoagulant.

# **eTable 2.** **Selected characteristics of the included studies**

| **Study** | **Data source** | **Study design** | **Study period** | **DOAC indication** | **Cohort size** | **Statistical analysis** | **Comparisons** | **Outcomes** |
| --- | --- | --- | --- | --- | --- | --- | --- | --- |
|  |  |  |  |  |  |  | **DOACs + PK-AA vs DOACs** |  |
| Chang^1^ | National Health Insurance database, Taiwan | Cohort | 2012-2016 | NVAF | 91,330 | IPTW | DOACs + amiodarone vs DOACs  DOACs + diltiazem vs DOACs  DOACs + dronedarone vs DOACs  DOACs + verapamil vs DOACs | Major bleeding |
| Chiou^2^ | Electronic health records from four hospitals, Taiwan | Cohort | 2011-2016 | AF | 2,388 | Cox regression | RIVA + amiodarone, propafenone, or dronedarone vs RIVA | Major bleeding, stroke/SE, all-cause mortality |
| Gandhi^3^ | Truven Health MarketScan claims data, US | Cohort | 2007-2017 | NVAF | 158,476 | PS stratification | APIXA + dronedarone vs APIXA  DABI + dronedarone vs DABI  RIVA + dronedarone vs RIVA | Major bleeding |
| Gronich^4^ | Clalit Health Services claims data, Israel | NCC | 2010-2020 | AF, DVT/PE | 89,284 | Conditional logistic regression with PS adjustment | DOACs + amiodarone vs DOACs  DOACs + diltiazem vs DOACs  DOACs + dronedarone vs DOACs  DOACs + verapamil vs DOACs | Major bleeding |
| Grymonprez^5^ | Nationwide claims data, Belgium | Cohort | 2013-2019 | Not reported | 193,072 | PS matching | DOACs + amiodarone vs DOACs  DOACs + diltiazem vs DOACs  DOACs + verapamil vs DOACs | Major bleeding, stroke/SE, all-cause mortality |
| Shurrab^6^ | Provincial claims data, Ontario | NCC | 2011-2018 | AF | 86,679 | Conditional logistic regression | DOAC + diltiazem vs DOACs | Major bleeding |
| Shurrab^7^ | Provincial claims data, Ontario | NCC | 2011-2018 | AF | 86,679 | Conditional logistic regression | DOACs + amiodarone vs DOACs | Major bleeding |
| Xu^8^ | Geisinger electronic health records, US | Cohort | 2010-2019 | AF | 4,544 | Cox regression | DOACs + diltiazem vs DOACs | Major bleeding |
|  |  |  |  |  |  |  | **DOACs + PK-AA vs DOACs + non-AA** |  |
| Hill^9^ | Provincial claims data, Ontario | Cohort | 2009-2016 | AF, DVT/PE, other | 94,866 | IPTW | DOACs + amiodarone vs DOACs + metoprolol  DOACs + diltiazem vs DOACs + amlodipine  DOACs + verapamil vs DOACs + amlodipine | Major bleeding |
| Komatsu^10^ | Inpatient health records, Japan | Cohort | 2011-2020 | Not reported | 617 | Cox regression | DOACs + verapamil vs DOACs + bepridil | Bleeding |
| Pham^11^ | Medicare, US | Cohort | 2010-2015 | NVAF | 4,869 / 5,017^*^ | IPTW | APIXA + verapamil or diltiazem vs APIXA + amlodipine  DABI + verapamil or diltiazem vs DABI + amlodipine  RIVA + verapamil or diltiazem vs RIVA + amlodipine  APIXA + verapamil or diltiazem vs APIXA + metoprolol  DABI + verapamil or diltiazem vs DABI + metoprolol  RIVA + verapamil or diltiazem vs RIVA + metoprolol | Major bleeding |
| Ray^12^ | Medicare, US | Cohort | 2012-2020 | NVAF | 204,155 | IPTW | DOACs + diltiazem vs DOACs + metoprolol | Major bleeding, stroke/SE |
| Teshima^13^ | Multiple integrated health care delivery systems, US | Cohort | Not reported | Not reported | 63,881 | Cox regression | APIXA + diltiazem or verapamil vs APIXA + metoprolol  DABI + diltiazem or verapamil vs DABI + metoprolol  RIVA + diltiazem or verapamil vs RIVA + metoprolol | Major bleeding, all-cause mortality |
| Wong^14^ | Clinical Practice Research Datalink Aurum, UK | Cohort | 2011-2019 | Not reported | 397,459 | PS | DOACs + amiodarone or diltiazem or verapamil vs DOACs + beta-blockers | Intracranial bleeding, gastrointestinal bleeding, other bleeding, stroke, all-cause mortality |
|  |  |  |  |  |  |  | **DOACs + PK-AA vs VKAs + PK-AA** |  |
| Friberg^15^ | Nationwide health registers, Sweden | Cohort | 2013-2016 | AF | 8,228 | PS matching | APIXA + dronedarone vs warfarin + dronedarone | Major bleeding, all-cause mortality |
| Fritz Hansson^16^ | Nationwide health registers, Sweden | Cohort | 2013-2018 | NVAF | 12,103 | PS matching | APIXA + amiodarone vs warfarin + amiodarone | Major bleeding, stroke/SE, all-cause mortality |
|  |  |  |  |  |  |  | **DOACs + PK-AA vs DOACs + non-PK-AA** |  |
| Ray^17^ | Medicare, US | Cohort | 2012-2018 | NVAF | 91,590 | IPTW | DOACs + amiodarone vs DOACs + flecainide or sotalol | Major bleeding, stroke/SE, all-cause mortality |

Abbreviations: DOACs, direct oral anticoagulants; PK-AA, pharmacokinetically-interacting antiarrhythmic drugs; VKAs, vitamin K antagonists; APIXA, apixaban; DABI, dabigatran; RIVA, rivaroxaban; NVAF, non-valvular atrial fibrillation; AF, atrial fibrillation; DVT, deep vein thrombosis; PE, pulmonary embolism; SE, systemic embolism; NCC, nested case-control; PS, propensity score; IPTW, inverse probability of treatment weighting.

^*^ There were 4,869 patients for the comparison DOACs + verapamil or diltiazem vs DOACs + amlodipine and 5,017 patients for the comparison DOACs + verapamil or diltiazem vs DOACs + metoprolol.

# **eTable 3. Selected clinical characteristics of patients in the included studies**

| **Study** | **DOACs** | **AAs** | **Mean age (years)^**^** | | **Female sex (%)** | | **Mean HAS-BLED score** | | **Mean CCI** | | **Prior ischemic stroke (%)** | | **Prior major bleeding (%)** | | **Chronic kidney disease (%)** | |
| --- | --- | --- | --- | --- | --- | --- | --- | --- | --- | --- | --- | --- | --- | --- | --- | --- |
| **DOACs + PK-AA vs DOACs alone** | | |  |  | |  | |  | |  | |  | |  | |  |
| Chang^1^ | APIXA, DABI, RIVA | Amiodarone, diltiazem, dronedarone, verapamil | 74.7 | | 55.8 | | 3.3 | | 2.4 | | 25.0 | | 12.3 | | NR | |
| Chiou^2*^ | RIVA | Amiodarone, dronedarone, propafenone | 73.1 - 74.1 | | 48.4 - 50.3 | | NR | | NR | | 20.3 - 30.3 | | 6.6 - 9.5 | | NR | |
| Gandhi^3^ | APIXA, DABI, RIVA | Dronedarone | 67.9 - 72.0 | | 57.1 - 62.4 | | NR | | NR | | 21.7 - 32.2 | | 4.9 - 9.8 | | 9.6 - 21.2 | |
| Gronich^4^ | APIXA, DABI, RIVA | Amiodarone, dronedarone, verapamil, diltiazem | 78.9 - 80.0 | | 50.3 | | NR | | NR | | NR | | NR | | 20.0 - 26.4 | |
| Grymonprez^5^ | APIXA, DABI, EDOXA, RIVA | Amiodarone, diltiazem, verapamil, | NR | | NR | | NR | | NR | | NR | | NR | | NR | |
| Shurrab^7*^ | APIXA | Amiodarone | 80.0 | | 51.7 | | NR | | 1.4 | | 8.7 | | NR | | 4.5 | |
| Shurrab^6*^ | APIXA | Diltiazem | 80.2 | | 51.7 | | NR | | 1.4 | | 8.7 | | NR | | 4.5 | |
| Xu^8^ | APIXA, DABI, RIVA | Diltiazem | 72 | | 55.5 | | 3.2 | | 2.1 | | 18.1 | | NR | | NR | |
| **DOACs + PK-AA vs DOACs + non-AA** | | |  |  | |  | |  | |  | |  | |  | |  |
| Hill^9^ | APIXA, DABI, RIVA | Amiodarone, diltiazem, verapamil | NR | | NR | | NR | | NR | | 1.0 - 3.1 | | NR | | NR | |
| Komatsu^10^ | APIXA, DABI, EDOXA, RIVA | Verapamil | 64.5 - 72.3 | | 55.6 – 77.8 | | NR | | NR | | 8.1 - 18.9 | | 4.2 - 15.0 | | NR | |
| Pham^11^ | APIXA, DABI, RIVA | Verapamil, diltiazem | NR | | 53.5 - 64.7 | | NR | | 1.5 - 1.9 | | 0.9 - 3.3 | | 7.1 - 12.4 | | NR | |
| Teshima^13^ | APIXA, DABI, RIVA | Verapamil, diltiazem | NR | | NR | | NR | | NR | | NR | | NR | | NR | |
| Ray^12^ | APIXA, RIVA | Diltiazem | 76.6 - 77.1 | | 40.7 - 49.6 | | 3.0 | | NR | | 6.8 - 8.1 | | 16.0 - 16.6 | | 3.0 - 3.4 | |
| Wong^14^ | APIXA, DABI, EDOXA, RIVA | Amiodarone, diltiazem, verapamil | NR | | 42.3 - 64.4 | | NR | | NR | | 11.3 - 15.2 | | 49.2 - 56.5 | | 1.6 - 3.0 | |
| **DOACs + PK-AA vs VKAs + PK-AA** | | |  |  | |  | |  | |  | |  | |  | |  |
| Friberg^15^ | APIXA | Dronedarone | 66.7 - 68.6 | | 56.2 – 57.2 | | 1.4 - 1.6 | | NR | | 4.3 - 6.3 | | 5.7 - 6.8 | | 1.0 - 1.9 | |
| Fritz Hansson^16*^ | APIXA | Amiodarone | 70.7 - 71.0 | | 66.2 - 66.6 | | NR | | NR | | 5.5 - 5.6 | | 9.9 | | 6.4 - 8.0 | |
| **DOACs + PK-AA vs DOACs + non-PK-AA** | | |  |  | |  | |  | |  | |  | |  | |  |
| Ray^17^ | APIXA, RIVA | Amiodarone | 74.6 - 77.3 | | 43.7 - 50.1 | | 2.8 - 3.2 | | NR | | 5.9 - 7.8 | | 15.6 - 18.0 | | 12.0 - 21.0 | |

Single values represent the values of the overall study cohort, ranges of values represent the values of different exposure groups.

^*^ Both studies applied a case control design nested within the same cohort of users of DOACs. Therefore, the reported clinical characteristics are identical.

^**^ Several studies reported age in pre-specified groups but not the mean value.

Abbreviations: DOACs, direct oral anticoagulants; PK-AA, pharmacokinetically-interacting antiarrhythmic drugs; VKAs, vitamin K antagonists; APIXA, apixaban; DABI, dabigatran; RIVA, rivaroxaban; EDOXA, edoxaban; HAS-BLED, hypertension, abnormal renal and liver function, stroke, bleeding, labile international normalized ratio, elderly, drugs or alcohol; NR, not reported; CCI, Charlson comorbidity index.

# **eTable 4. Different forms of bias in selection of participants into study**

| **Study** | **Exclusion of persons or person-time after cohort entry** | **Depletion of susceptibles due to inclusion of prevalent users** | **Immortal time bias** | **Informative censoring** | **Depletion of susceptibles due to design aspects** | **Overall** |
| --- | --- | --- | --- | --- | --- | --- |
| Chang^1^ | Yes | Yes | No | No | Yes | Critical |
| Chiou^2^ | Yes | Yes | No | Yes | No | Critical |
| Friberg^15^ | No | Yes | No | Yes | No | Serious |
| Fritz Hansson^16^ | No | Yes | No | Yes | No | Serious |
| Gandhi^3^ | No | Yes | No | Yes | No | Serious |
| Gronich^4^ | No | No | No | No | Yes | Moderate |
| Grymonprez^5^ | No | Yes | No | No | No | Moderate |
| Hill^9^ | No | No | No | Yes | No | Moderate |
| Komatsu^10^ | No | Yes | No | Yes | No | Serious |
| Pham^11^ | No | Yes | No | No | No | Moderate |
| Ray^17^ | No | No | No | No | No | Low |
| Ray^12^ | No | No | No | No | No | Low |
| Shurrab^7^ | No | Yes | No | No | Yes | Serious |
| Shurrab^6^ | No | Yes | No | No | Yes | Serious |
| Teshima^13^ | No | Yes | No | Yes | No | Serious |
| Wong^14^ | No | Yes | No | No | Yes | Serious |
| Xu^8^ | No | Yes | No | No | No | Moderate |

3 different types of bias in selection of participants into study 🡪 overall **critical** risk of selection bias

2 different types of bias in selection of participants into study 🡪 overall **serious** risk of selection bias

1 different type of bias in selection of participants into study 🡪 overall **moderate** risk of selection bias

0 different types of bias in selection of participants into study 🡪 overall **low** risk of selection bias

# **REFERENCES**

1. Chang SH, Chou IJ, Yeh YH, et al. Association Between Use of Non-Vitamin K Oral Anticoagulants With and Without Concurrent Medications and Risk of Major Bleeding in Nonvalvular Atrial Fibrillation. JAMA 2017;318(13):1250-1259.

2. Chiou WR, Lin PL, Huang CC, et al. Rhythm control without catheter ablation may have benefits beyond stroke prevention in rivaroxaban-treated non-permanent atrial fibrillation. Sci Rep 2022;12(1):3745.

3. Gandhi SK, Reiffel JA, Boiron R, Wieloch M. Risk of Major Bleeding in Patients With Atrial Fibrillation Taking Dronedarone in Combination With a Direct Acting Oral Anticoagulant (From a U.S. Claims Database). Am J Cardiol 2021;159:79-86.

4. Gronich N, Stein N, Muszkat M. Association Between Use of Pharmacokinetic-Interacting Drugs and Effectiveness and Safety of Direct Acting Oral Anticoagulants: Nested Case-Control Study. Clin Pharmacol Ther 2021;110(6):1526-1536.

5. Grymonprez M, Carnoy L, Capiau A, et al. Impact of P-glycoprotein and CYP3A4-interacting drugs on clinical outcomes in patients with atrial fibrillation using non-vitamin K antagonist oral anticoagulants: a nationwide cohort study. Eur Heart J Cardiovasc Pharmacother 2023;9(8):722-730.

6. Shurrab M, Jackevicius CA, Austin PC, et al. Association between concurrent use of diltiazem and DOACs and risk of bleeding in atrial fibrillation patients. J Interv Card Electrophysiol 2023;66(3):629-635.

7. Shurrab M, Jackevicius CA, Austin PC, et al. Association Between Concurrent Use of Amiodarone and DOACs and Risk of Bleeding in Patients With Atrial Fibrillation. Am J Cardiol 2023;186:58-65.

8. Xu Y, Chang AR, Inker LA, McAdams-DeMarco M, Grams ME, Shin JI. Concomitant Use of Diltiazem With Direct Oral Anticoagulants and Bleeding Risk in Atrial Fibrillation. J Am Heart Assoc 2022;11(14):e025723.

9. Hill K, Sucha E, Rhodes E, et al. Amiodarone, Verapamil, or Diltiazem Use With Direct Oral Anticoagulants and the Risk of Hemorrhage in Older Adults. CJC Open 2022;4(3):315-323.

10. Komatsu Y, Yodoshi M, Takegami M, Yokoyama S, Hosomi K. Association between hemorrhage and direct oral anticoagulants in combination with verapamil: Analysis of Japanese Adverse Drug Event Report database and electronic medical record data. Int J Clin Pharmacol Ther 2023;61(4):148-158.

11. Pham P, Schmidt S, Lesko L, Lip GYH, Brown JD. Association of Oral Anticoagulants and Verapamil or Diltiazem With Adverse Bleeding Events in Patients With Nonvalvular Atrial Fibrillation and Normal Kidney Function. JAMA Netw Open 2020;3(4):e203593.

12. Ray WA, Chung CP, Stein CM, et al. Serious Bleeding in Patients With Atrial Fibrillation Using Diltiazem With Apixaban or Rivaroxaban. JAMA 2024;331(18):1565-1575.

13. Teshima SSM, Casanova EM, Drees ET, et al. Safety of direct oral anticoagulant use in patients taking verapamil or diltiazem. Heart Rhythm 2023;20(12):1793-1795.

14. Wong AYS, Warren-Gash C, Bhaskaran K, et al. Potential interactions between medications for rate control and direct oral anticoagulants: Population-based cohort and case-crossover study. Heart Rhythm 2024.

15. Friberg L. Safety of apixaban in combination with dronedarone in patients with atrial fibrillation. Int J Cardiol 2018;264:85-90.

16. Fritz Hansson A, Modica A, Renlund H, Christersson C, Held C, Batra G. Major bleeding in patients with atrial fibrillation treated with apixaban versus warfarin in combination with amiodarone: nationwide cohort study. Open Heart 2024;11(1)

17. Ray WA, Chung CP, Stein CM, et al. Risk for Bleeding-Related Hospitalizations During Use of Amiodarone With Apixaban or Rivaroxaban in Patients With Atrial Fibrillation : A Retrospective Cohort Study. Ann Intern Med 2023;176(6):769-778.
